# Application of bacteriophages in the prevention and control of bacterial infectious diseases in animals

**DOI:** 10.3389/fmicb.2026.1851321

**Published:** 2026-08-05

**Authors:** Junyao Li, Huan Zhang, Haihua Yu, Peiyi Liang, Songbai Xu, Lili Zhong, Xiying Fu, Yifan Zhang, Yicun Wang

**Affiliations:** 1 Department of Respiratory and Critical Care Medicine, The Second Hospital of Jilin University, Changchun, China; 2 Nephrology Department, Affiliated Hospital of Changchun University of Chinese Medicine, Changchun, China; 3 Physical Examination Center, The Second Hospital of Jilin University, Changchun, China; 4 Department of Medical Research Center, Second Hospital of Jilin University, Changchun, China; 5 Department of Neurosurgery, First Hospital of Jilin University, Changchun, China; 6 Department of Endocrinology, Second Hospital of Jilin University, Changchun, China; 7 Department of Breast Surgery, Second Hospital of Jilin University, Changchun, China

**Keywords:** animal health, antimicrobial resistance, engineered phage, mucosal immunity, phage therapy, phage vaccine, synthetic biology

## Abstract

The global spread of antimicrobial resistance (AMR) has intensified the search for alternatives to conventional antibiotics in animal production systems. Bacteriophages can be engineered beyond narrow-spectrum antibacterial agents into multifunctional biological platforms that integrate direct killing, immune modulation, and antigen delivery. We summarize recent advances across livestock, poultry, and aquaculture, delineating mechanistic distinctions between lytic phage therapy, phage display-derived interventions, and engineered platforms including CRISPR-Cas-enabled theranostic systems. However, as detailed below, most evidence remains preclinical, and translational gaps are substantial. Unlike prior descriptive reviews, we analyze translational bottlenecks—host range constraints, pharmacokinetic limitations, regulatory fragmentation—and assess the existing research evidence for claimed advantages such as microbiota preservation and biofilm penetration while upfront acknowledging inconsistent experimental outcomes and inherent application limitations behind these beneficial effects. We conclude that realizing phages’ therapeutic potential in veterinary medicine requires coordinated progress in synthetic biology, scalable manufacturing, and regulatory harmonization within a One Health framework.

## 1 Introduction

Global animal production systems, while meeting protein demand, have established substantial reservoirs of pathogenic microorganisms. Bacterial pathogens including *Escherichia coli*, *Salmonella enterica*, *Staphylococcus aureus*, and *Vibrio* spp. cause significant morbidity and mortality in livestock, poultry, and aquaculture (Elbehiry and Marzouk, 2025), with annual economic losses exceeding USD 20 billion worldwide. These animal-associated pathogens also serve as reservoirs of antibiotic resistance genes transmissible to humans through food chains and environmental pathways (Kiskinis et al., 2024). Decades of antimicrobial use in livestock have progressively eroded therapeutic efficacy; surveillance data indicate that >35% of bacterial isolates from livestock and poultry exhibit multidrug resistance (Li J. et al., 2025).

Beyond resistance, conventional antibiotics disrupt gut microbiota essential for nutritional and immune homeostasis (Ramirez et al., 2020; Cusumano et al., 2025), and show limited efficacy against biofilm-embedded pathogens or intracellular bacteria such as *Salmonella* (Bai et al., 2023; Thakur et al., 2019; Liu et al., 2024). Prophylactic vaccines offer partial protection but face constraints in development timelines, mucosal immunity induction, and adaptability to emerging pathogens (Tobias et al., 2024).

Bacteriophages—viruses that specifically infect bacteria—present a mechanistically distinct alternative. With an estimated 10 (Xie et al., 2024) phage particles globally, their co-evolution with bacterial hosts has shaped highly specific infection mechanisms. Relative to chemical antibiotics, phages offer several advantages: minimal mammalian toxicity, high targeting precision with presumably limited disruption of commensal microbiota, self-amplification at infection sites, enhanced biofilm penetration via phage-encoded depolymerases, and genetic editability (Peng et al., 2025). Nevertheless, accumulating evidence confirms such benefits cannot be universally recapitulated under complex *in vivo* scenarios; multiple limiting factors compromise phage-mediated microbiota homeostasis and pathogen elimination under field conditions.

Recent advances in genomics and synthetic biology have transformed phages from natural “bacterial predators” into programmable platforms for antigen delivery, genome editing, and immune modulation (Alessa et al., 2025). This review systematizes the evolutionary progression of phage therapeutics from simple antibacterials to multifunctional anti-infection platforms, objectively analyzes underlying molecular mechanisms, assesses published data with due consideration of experimental constraints and variable *in vivo* efficacy, and delineates developmental routes to inform veterinary translational practice.

## 2 Biological characteristics of bacteriophages

### 2.1 Lytic cycle and host specificity

Bacteriophages are viruses that specifically infect and replicate within bacterial cells, ubiquitously distributed in soil, aquatic ecosystems, and animal gastrointestinal tracts (Brown et al., 2022). Host specificity is mediated by tail fibers or spike proteins that recognize bacterial surface receptors such as lipopolysaccharides in Gram-negative bacteria or teichoic acids in Gram-positive bacteria (Taslem Mourosi et al., 2022). The host range of bacteriophages is determined by receptor-binding proteins. Several mechanisms contribute to host range variability, including key amino acid mutations that directly alter the interaction network at the receptor-binding interface (He et al., 2024); modular domain recombination that acquires novel specificities through swapping C-terminal recognition domains (Sørensen et al., 2024); conformational activation whereby initial binding induces downstream functional exposure (d'Acapito et al., 2025); and branched receptor-binding complexes (e.g., in Ackermannviridae) that enable multi-spike cooperative recognition to evade bacterial mutations (Sørensen et al., 2024). Together, these mechanisms empower bacteriophages to flexibly expand their host range through microevolution and macroevolution. The lytic cycle proceeds through five conserved stages: adsorption, genome injection, biosynthesis, virion assembly, and lysis via phage-encoded endolysins. This targeted mechanism enables selective pathogen elimination while preserving commensal flora—a key distinction from broad-spectrum antibiotics (Gundersen et al., 2023; Getz et al., 2025).

### 2.2 Phage display technology

Phage display technology enables construction of diverse genetic libraries by fusing exogenous peptide- or protein-coding DNA sequences to genes encoding phage coat proteins—pIII of filamentous phage M13 or gp23 of bacteriophage T4—generating libraries containing billions of unique variants (Hutchings and Sato, 2024). Each phage particle physically links displayed molecule (“phenotype”) to its encoding DNA (“genotype”), enabling high-throughput screening for target binders. This technology has been applied extensively in biomedical research; for instance, filamentous phage-based vaccines displaying *Candida albicans* epitopes elicited protective immunity in murine fungal infection models (Wang et al., 2006; Huai et al., 2016), while engineered phages have served as tumor-targeted delivery vehicles (Li H. R. et al., 2025; Islam et al., 2023). The application of phage display to vaccine development is further discussed in Section 3.

### 2.3 Advantages for infection control

**Figure 1:**
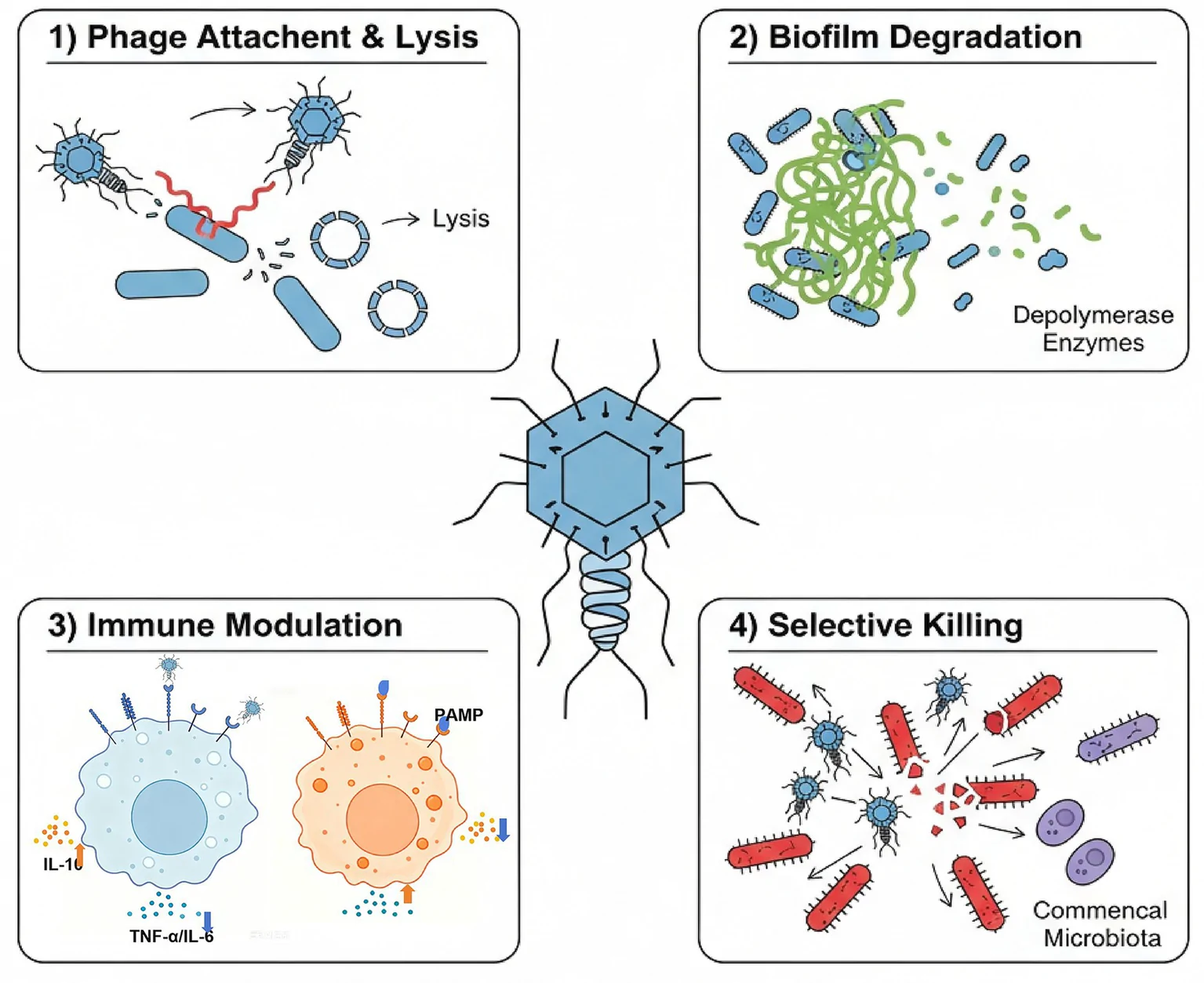
Mechanistic framework of bacteriophage-based control of bacterial infections bacteriophages control bacterial infections through multiple complementary mechanisms. (1) Receptor-mediated lysis—phages replicate within and lyse specific bacteria, enabling self-amplifying killing. (2) Biofilm disruption—phage depolymerases degrade the biofilm matrix, enhancing bacterial access. (3) Immune activation—T4 phage activates immunity and modulates cytokine secretion to improve the immune dysregulation induced by PAMPs released from disrupted bacteria. (4) Selective targeting—high host specificity allows pathogen elimination while preserving commensal microbiota. These mechanisms position phages as precision antimicrobials integrating direct killing and immune modulation.

Three attributes distinguish phages from conventional antimicrobials. First, recognition specificity shaped by billions of years of co-evolution with bacterial hosts surpasses that of many synthetic targeting systems (Kortright et al., 2019). Second, phages self-amplify at infection sites, dynamically maintaining local therapeutic concentrations without repeated dosing (Hibstu et al., 2022). Third, bacterial lysis releases pathogen-associated molecular patterns (PAMPs), while phage genomic DNA stimulates innate immune signaling, producing combined bactericidal and immunomodulatory effects (Kortright et al., 2019; Gembara and Dąbrowska, 2024) (Figure 1). These properties position phage-based strategies for addressing persistent infections including those caused by antibiotic-resistant pathogens, biofilm-associated diseases, and conditions requiring effective mucosal immune protection (see Table 1).

**Table 1:** Mechanisms of action of different types of bacteriophages in the prevention and treatment of animal bacterial infections.

Application orientation	Representative bacteriophage types	Core characteristics	Typical application cases	References
Direct lytic action	Virulent bacteriophages (Myoviridae: vB_SauM_JDYN, vB_SauM_JDF86; Siphoviridae/Podoviridae: *Vibrio*-specific bacteriophages)	Lytic-only, no host genome integration; high host specificity for pathogenic bacteria lysis; non-toxic to beneficial flora.	Prevention and control of bovine mastitis (caused by *Staphylococcus aureus*), prevention and control of shrimp *Vibrio*sis, treatment of pathogenic *E. coli* disease in chicks	Guo et al. (2024), Moon et al. (2025), Moreno et al. (2025)
Lytic and vector functions	Virulent bacteriophages (T4, T7), modified temperate bacteriophages (λ bacteriophage)	Strong natural lytic activity; high genomic manipulability via non-essential regions; dual lytic-vector functions post-engineering.	SMART technology splits T7 genome for parallel editing, enabling heterologous lyase loading and cross-species lysis; T7-DspB reduces biofilm bacteria by ~4.5 logs via dispersin B expression; CRISPR-Cas9 introduces Aiia/DP into PaGZ-1 for dual biofilm inhibition and disruption.	Lu and Collins (2007), Eghbalpoor et al. (2024), Zhang H. et al. (2025)
Vector-only function	Filamentous bacteriophages (M13, fd), modified defective temperate bacteriophages	Low lytic activity, stable genome for efficient foreign protein display, high targeted delivery, and controllable immunogenicity.	Fd bacteriophage enables targeted antigen delivery with TLR9-mediated adjuvanticity for antibacterial immunity.	Sartorius et al. (2015), Łobocka et al. (2021), Li Y. et al. (2025)

## 3 Direct phage lytic therapy: precision antimicrobial action

### 3.1 Applications in major bacterial diseases of production animals

Direct lytic therapy relies on virulent phages to eliminate bacteria via receptor-dependent lysis, with Table 2 cataloging relevant animal applications by pathogen, administration route, efficacy and resistance profile for inter-disease comparison. Collation of Table 2 data across poultry, swine, cattle and aquaculture reveals optimized phage cocktails efficiently eradicate pathogens with little damage to commensal microbiota, an outcome conflicting with Ref. (von Strempel et al., 2023) may due to divergent animal models and screening designs: test phages herein are preselected to avoid attacking beneficial microbes, while those in Ref. (von Strempel et al., 2023) were intentionally engineered to lyse essential gut commensals. Poultry Colibacillosis: Avian pathogenic *E. coli* (*APEC*) causes severe septicemia. Phage cocktails—mixtures of multiple phages with complementary host ranges—address strain diversity. Oral administration of a cocktail via drinking water reduced mortality in challenged chicks and decreased intestinal *APEC* loads without adversely affecting beneficial genera including *Lactobacillus* and Bacteroides (Upadhaya et al., 2021). Mechanistically, the cocktail’s efficacy derives from recognition of distinct *APEC* receptors (lipopolysaccharide, OmpC), enabling synergistic lysis while avoiding host competition that can limit single-phage efficacy.

**Table 2:** Application of bacteriophage direct lytic action in different animal bacterial infections.

Disease name	Host animal	Pathogenic agent	Bacteriophage type	Application method	Core mechanism	Core prevention and control effect	Research/application stage	References
Avian pathogenic *E. coli* (*APEC*) disease	Laying hens	Avian Pathogenic *E. coli* (*APEC*)	Phage cocktail consisting of 3 specific lytic bacteriophages	Oral administration via drinking water	Three phage genera target distinct *APEC* receptors (LPS, OmpC, etc.) for synergistic lysis, activate TLR4-NF-κB innate immunity, distribute systemically for organ protection, and preserve cecal microbiota.	Mortality reduced from 20% to 0, morbidity down 30%, no macroscopic lesions, lower histopathology scores (*p* = 0.006 in spleen), systemic phage distribution (1.3–2.1 log PFU/g), and no disruption of cecal microbiota.	In vivo preclinical efficacy study	Gohar et al. (2026)
Bovine mastitis	Mouse model + Dairy cows	*Staphylococcus aureus* (including *MRSA*)	Phage cocktail PHC-1	Intramammary injection	PHC-1: 3 Myoviridae phages, synergistic bacteriolysis, minimal resistance, broad stability. Krömker: proprietary cocktail, 5 × IMAM infusions, broad host range, reduced escape risk.	81.3% bacteriological cure (13/16) vs. 28.6% spontaneous cure (2/7) in naturally infected dairy cows (*p* = 0.026); 5 × IMAM infusions well-tolerated, no systemic side effects.	Preclinical + Pilot clinical	Guo et al. (2024), Krömker et al. (2026)
Avian necrotic enteritis	Broiler Chicks	*Clostridium perfringens*	Lytic (virulent) phage, Gregsiragusavirus genus	Oral / Feed addition (combined with probiotic *Pediococcus pentosaceus*)	Virulent phage SLAM_phiCP2S1 directly lyses *C. perfringens*; plus probiotic *P. pentosaceus* restores gut microbial homeostasis.	partial protection. Phage + probiotic: most effective—reduced *C. perfringens* burden to near control levels, preserved growth and intestinal integrity, restored gut microbial homeostasis.	Preclinical proof-of-concept	Choi et al. (2026)
Poultry salmonellosis	Breeding eggs	*Salmonella*	Lytic phage vB_SenM_BP13076	Direct egg surface application	BP13076 (Caudoviricetes): 5 min latency, 105 PFU/cell burst, lyses 66/68 *Salmonella* strains, no virulence/AMR/lysogeny genes	MOI 100–1,000 on eggs → significant *Salmonella* reduction vs. controls	pre-field trial	Wang et al. (2025)
Poultry salmonellosis	Broiler chickens + Poultry	*S. enteritidis* + MDR *S. enteritidis*	Lytic cocktail (3 phages, Jerseyvirus) + Virulent cocktail (3 phages, Caudovirales)	Oral/water + Oral (pre/co-infection)	UPWr_S134: Broad host range, robust pH/thermal stability, GI survival, no virulence/AMR/lysogeny genes. GRN: Short latency, broad stability, GRNsp8 expands host range, no virulence/AMR/lysogeny gene	UPWr_S134: ↓Liver/spleen/ceca counts; delayed symptoms. GRN: ↓Cecal colonization; ↓IL-6/IFN-γ/IL-1β	Preclinical in vivo	Li et al. (2022), Kuźmińska-Bajor et al. (2023)
Porcine salmonellosis	Swine	Multidrug-resistant *Salmonella* strains	Lytic phage cocktail SPFM10-SPFM14	Oral administration	6 lytic Myoviridae phages (SPFM2/4/10/14/17/19) target *Salmonella* via LPS O-antigen; burst size 164 PFU/cell, latency 20-30 min; broad pH/temp stability	Larvae survival 90% vs. 3% (72 h); 65% of treated larvae cleared *Salmonella* vs. 100% positive in controls; *in vitro*: ~2 × 10^3^ CFU/mL reduction in 2 h	Preclinical in vivo	Thanki et al. (2022)

Poultry Salmonellosis: Oral administration of the UPWr_S134 phage cocktail significantly reduced *Salmonella Enteritidis* load in internal organs of infected broilers (Kuźmińska-Bajor et al., 2023). Similarly, a cocktail of three virulent phages (GRNsp6, GRNsp8, GRNsp51) administered before or alongside *Salmonella* challenge significantly reduced intestinal colonization and pro-inflammatory cytokine expression (Li et al., 2022). Spray application of phage vB_SPuM_SP02 eradicated *Salmonella Typhimurium* from contaminated eggshells, increasing hatching and chick survival rates (Wang et al., 2024). *In vitro* studies reveal protective mechanisms: phage pSal-4 reduced adhesion and invasion of *Salmonella Enteritidis* into chicken intestinal epithelial cells while alleviating inflammation and restoring tight junction protein expression (Occludin, Claudin-1, ZO-1) (Xie et al., 2024).

Porcine Salmonellosis: In recent years, remarkable progress has been made in phage therapy research against swine salmonellosis. A phage cocktail composed of six lytic phages (SPFM2, SPFM4, SPFM10, SPFM14, SPFM17, SPFM19), which were isolated and characterized from swine-associated multidrug-resistant *Salmonella* strains, markedly increased the 72-h survival rate of infected *Galleria mellonella* larvae from 3 to 90% (Thanki et al., 2022). Moreover, *Salmonella* was eliminated below the detection limit in 65% of treated larvae. These findings provide preliminary theoretical support for subsequent efficacy verification of oral phage therapy in pigs.

Bovine Mastitis: Recent advances have demonstrated the progressive development of phage cocktail therapy against *Staphylococcus aureus*-induced bovine mastitis. From the validation of the synergistic bacteriolytic effect of PHC-1 in a mouse mastitis model, to the first randomized controlled trial confirming the clinical efficacy of a proprietary phage cocktail in naturally infected dairy cows, phage therapy for *S. aureus*-induced bovine mastitis has advanced from preclinical proof-of-concept to clinical pilot validation, suggesting its potential as an antibiotic alternative worthy of further investigation (Krömker et al., 2026; Guo et al., 2024).

Aquaculture Infections: In shrimp farming, *Vibrio* outbreaks cause catastrophic losses (Xiao et al., 2017). A broad-host-range phage cocktail administered directly into shrimp aquaculture water selectively suppressed *Vibrio* populations and dynamically regulated microbial community structure, significantly increasing larval survival (Lomelí-Ortega et al., 2023).

### 3.2 Mechanisms of action against multidrug-resistant bacteria

Phage lytic activity operates through mechanisms fundamentally distinct from antibiotic resistance pathways (efflux pumps, drug-modifying enzymes, target site mutations). Consequently, phages retain antibacterial activity against pathogens refractory to conventional agents.

Phage–antibiotic combination therapy can yield synergistic outcomes. Sub-inhibitory concentrations of certain antibiotics enhance phage activity (Luo et al., 2023). Sub-MIC ciprofloxacin induces filamentation and cell wall stress in *E. coli*, exposing additional phage receptors and sensitizing the cell wall to phage endolysins, producing synergistic killing *in vitro* and in animal infection models (Bulssico et al., 2023; Butler et al., 2023; Kim et al., 2018). This synergy suggests phages may be integrated into combination regimens rather than simply replacing antibiotics. However, the phage-antibiotic synergism faces multiple challenges, including the lack of standardized methodologies, time-consuming personalized preparation, imperfect large-scale production and regulatory frameworks, as well as inherent biological limitations of phages such as narrow host spectrum, poor stability and easy emergence of bacterial resistance (Kline et al., 2025; Ayele et al., 2026).

To address these inherent limitations,the most sophisticated approach uses CRISPR-Cas-engineered bacteriophages. In this strategy, phage genomes carry CRISPR-Cas systems programmed to cleave essential bacterial genes or specific antibiotic resistance determinants (Lee et al., 2025; Hill and Hatoum-Aslan, 2024). Such engineered phages exert dual action: conventional lytic killing plus CRISPR-Cas-mediated genomic disruption. In a murine intestinal colonization model, CRISPR-phage reduced target *E. coli* populations by 3–4 log units with minimal impact on non-target microbiota, achieving unprecedented precision (Gencay et al., 2024). However, this system is constrained by the narrow host range of phage vectors leading to low delivery efficiency, *in vivo* delivery barriers, off-target effects, as well as complex production processes that hinder scalable manufacturing and severe deficiencies in clinical translation (Tsolakidou, 2025).

## 4 Bacteriophages as vaccine vectors: immunostimulatory nanoscale scaffolds

Beyond direct lytic activity, phages serve as vaccine vectors. Their nanoscale architecture, efficient genome packaging, and intrinsic immunostimulatory properties offer potential advantages over traditional subunit or inactivated vaccines, though quantitative benchmarking against these platforms remains limited.

### 4.1 Molecular basis of immune activation

**Figure 2:**
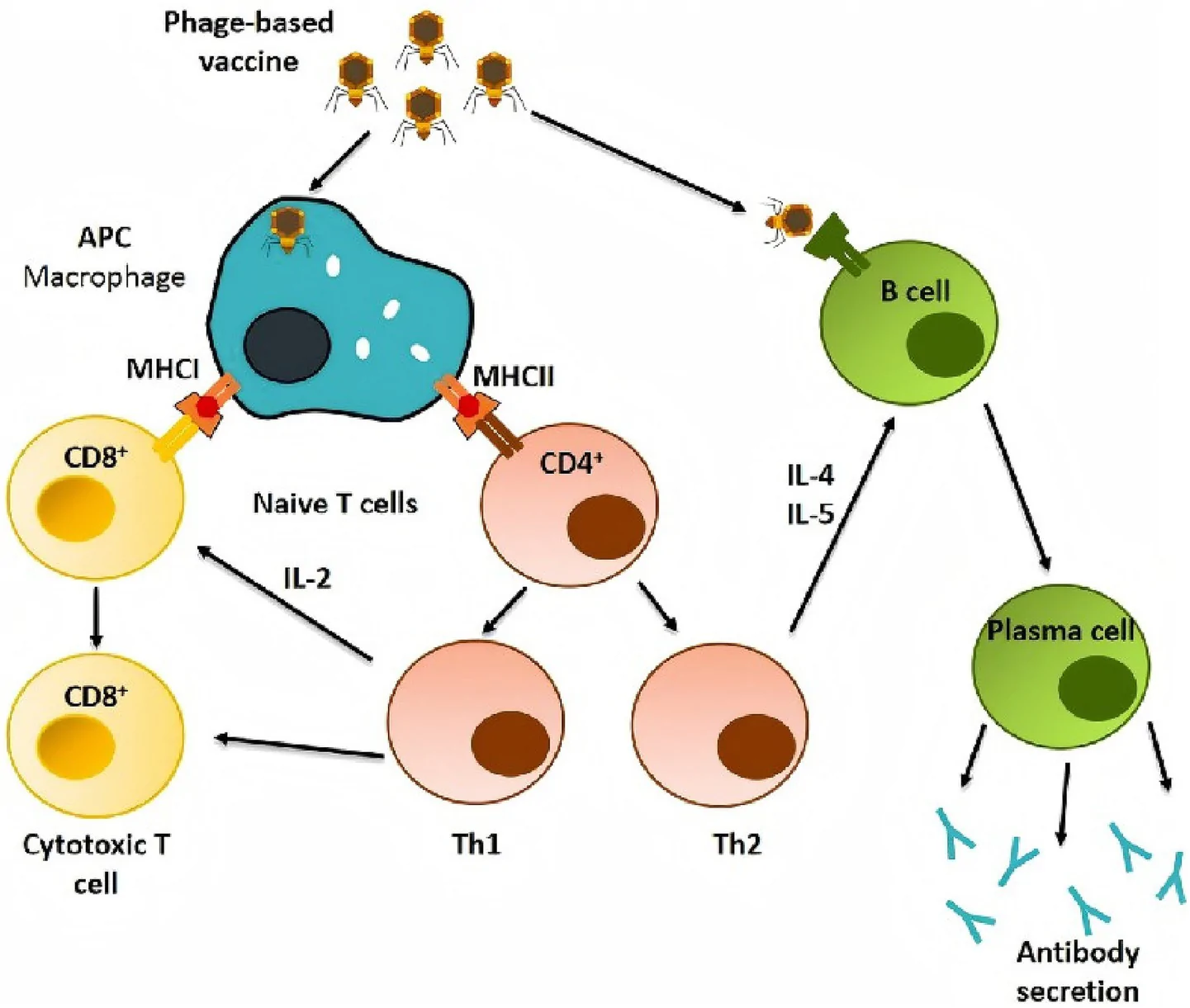
Phage-based antigen delivery and dual MHC pathway activation (González-Mora et al., 2020). Dual MHC pathway activation. Phage-displayed antigens are taken up by APCs, processed, and presented via MHC class II (CD4^+^ T helper cells) and cross-presentation via MHC class I (cytotoxic CD8^+^ T cells), driving humoral and cellular immunity. Reprinted from Figure 3 in González-Mora et al. (2020) under CC BY 4.0.

Phage immunogenicity derives from three mechanisms. First, phage genomes are rich in unmethylated CpG motifs recognized by Toll-like receptor 9 (TLR9) in plasmacytoid dendritic cells, triggering type I interferon and pro-inflammatory cytokine responses that polarize adaptive immunity toward a Th1 phenotype (Mohammad Hasani et al., 2023; Chuang et al., 2020). Second, the repetitive high-density array of capsid proteins cross-links B cell receptors, driving T cell-independent IgM responses that can be engineered to elicit T cell-dependent, high-affinity IgG (Samoylova et al., 2017; Yin et al., 2013). Third, via phage display, antigen genes fused to coat protein genes produce high-density ordered antigen presentation on virion surfaces (Hess and Jewell, 2020). This quasi-crystalline presentation mimics natural pathogens, promoting uptake by antigen-presenting cells (APCs). Internalized antigens are processed and presented via MHC class II pathways (activating CD4^+^ T helper cells) and, through cross-presentation, MHC class I pathways (activating CD8^+^ cytotoxic T cells) (Blander, 2016; de Vries et al., 2021; Sengupta et al., 2024) (Figure 2).

### 4.2 Evidence for vaccine platform performance

In studies on avian influenza and Newcastle disease, researchers have developed multiple vaccine strategies based on viral antigen epitopes. M13 phage displaying HA2 epitopes can induce specific IgY antibody production in chickens (Lotfi et al., 2026). The immunoinformatics-designed multi-epitope vaccine (NFAF) is capable of stimulating chicken PBMCs and eliciting Th1/Th2-type cytokines (Ji et al., 2025). The NDV-vectored H7N9 vaccine confers full challenge protection by activating the complement system (Hu et al., 2024). Nevertheless, these vaccines have respective limitations, including failure to markedly reduce viral shedding, lack of viral challenge verification, and unassessed T cell immune responses.

In studies on porcine epidemic diarrhea virus and foot-and-mouth disease virus, recombinant phage display systems can effectively induce specific humoral and/or cellular immune responses (Li Y. et al., 2025; Chen et al., 2024), demonstrating their broad applicability as a novel vaccine delivery platform.

### 4.3 Comparison with current veterinary vaccine platforms

Compared with conventional veterinary vaccine platforms, phages serve as vaccine vectors with high biosafety, favorable stability and flexible genetic engineering modification capability. Although traditional inactivated vaccines and subunit vaccines feature high safety, they generally suffer from weak immunogenicity, adjuvant dependence and the need for multiple immunizations (Honda-Okubo et al., 2021). Live attenuated vaccines can elicit robust immune responses, yet they carry the risk of virulence reversion (Wiebe et al., 2025). In contrast, phages can deliver antigens at high density by displaying pathogenic epitopes on their surface. Their capsid proteins and CpG-rich DNA are able to activate innate immunity, thereby improving antigen presentation efficiency and potentiating humoral, cellular and mucosal immune responses (Zhu et al., 2024).

Nevertheless, most current studies remain confined to laboratory settings and small animal models. Their long-term protective efficacy in livestock animals and industrialized application still require further verification.

## 5 Engineered phage platforms: integrating therapeutic and prophylactic functions

Synthetic biology enables deliberate redesign of phage genomes, creating agents that transcend the treatment–prevention dichotomy. “Theragnostic” phages perform immediate antibacterial therapy while training host immune systems for long-term protection.

### 5.1 Design strategies

#### 5.1.1 “Lysis-presentation” dual-function phages

This strategy preserves the lytic machinery of virulent phages (e.g., T7) while incorporating genes encoding pathogen-specific antigens fused to capsid protein genes (Yue et al., 2022). Upon infecting target bacteria, the engineered phage executes its lytic cycle, directly killing the pathogen. Progeny virions carry foreign antigens on their surface; these particles, along with bacterial antigens from lysate, are taken up by APCs at infection sites, priming adaptive immune responses against the pathogen (González-Mora et al., 2020).

#### 5.1.2 “Lysogeny-regulation” vectors

This strategy exploits temperate phages (e.g., *λ*) capable of lysogeny—integrating their genome into host chromosomes as prophages. Engineered temperate phages can remain latent until specific triggers (bacterial quorum-sensing molecules, external chemical inducers) initiate the lytic cycle. During lysogenic phases, these phages continuously express and secrete therapeutic molecules including neutralizing antibody fragments targeting bacterial toxins, immunomodulatory cytokines (e.g., IFN-*γ*) to reshape local immune microenvironments (Cianci et al., 2025), or biofilm-degrading enzymes (Islam et al., 2024). This provides sustained localized therapy that escalates to full lytic attack upon sensing increased bacterial burden.

### 5.2 Proof-of-concept applications

T2 bacteriophages displaying *Salmonella typhimurium* antigens (FliC and FljB segments) administered orally to mice rapidly reduced *Salmonella* and subsequently induced high-titer serum anti-*Salmonella* IgG and intestinal sIgA (Synnott et al., 2009). In mouse models of pulmonary infection, traumatic infection and sepsis, engineered phages can reduce bacterial load, alleviate inflammatory responses and improve survival rates (Rahimi et al., 2023; Park et al., 2024; Cui et al., 2023). For intracellular pathogens including *Salmonella*, phages armed with cell-penetrating peptides on capsids could lyse extracellular bacteria, escape phagosomes, and lyse intracellular *Salmonella*—a feat impossible for conventional antibiotics or wild-type phages (Zhao et al., 2023). Moreover, engineered phages can serve as CRISPR-Cas delivery platforms, enabling precise killing of *S. aureus* and significantly reducing bacterial load and inflammatory levels in mastitis models (Garmendia-Antoñana et al., 2026). Compared to conventional antibiotics, this strategy offers advantages such as strong targeting specificity, reduced disruption of the normal microbiota, and lower risk of promoting resistance dissemination, collectively highlighting the potential of engineered phages in precision anti-infective therapy.

### 5.3 Persistent challenges

Although engineered phages have shown promising application prospects in experimental research, their clinical and industrial transformation still faces numerous regulatory and safety challenges. At present, most studies lack long-term toxicological evaluation, systematic immunogenicity analysis and stability assessment in complex biological environments. As replicable biological agents, engineered phages may trigger potential risks *in vivo*, including host immune clearance (Le et al., 2025), horizontal gene transfer and intestinal flora imbalance (Youssef et al., 2025), thus establishing a more systematic biosafety evaluation system is urgently needed.

In addition, engineered phages also encounter obstacles in production and supervision. For instance, endotoxins, bacterial proteins and host DNA may remain during purification, impairing the safety and repeatability of phage preparations (Saavedra et al., 2025). Currently, unified international regulatory standards for engineered phages have not been fully established, and mature specifications are still absent in terms of drug classification, quality control, clinical approval and environmental release management.

## 6 Indirect applications: phage-derived peptides and antibodies

Beyond whole virions, phage display enables high-throughput discovery of bioactive molecules—peptides, antibody fragments—that can be deployed independently as antimicrobials, virulence inhibitors, or diagnostics.

### 6.1 Identification of protective epitopes

Phage-displayed random peptide libraries screened with specific monoclonal antibodies can identify conserved B-cell epitopes. A study using this approach identified a conserved epitope on *Streptococcus dysgalactiae* GapC protein; the corresponding synthetic peptide induced protective immune responses in immunized mice (Zhang et al., 2015).

### 6.2 Therapeutic peptides and antibodies

In aquaculture, two peptides targeting *Vibrio mimicus* OmpU exhibited adhesion antagonistic activity (Qi et al., 2016). Phage antibody libraries (scFv, Fab) enable rapid isolation of high-affinity binders (Liu et al., 2024). For bovine mastitis caused by *S. aureus*, scFv antibodies screened from phage libraries exhibited strong binding activity and effectively inhibited bacterial growth (Wang et al., 2019).

### 6.3 Advantages and translational hurdles

The core advantages of phage-derived molecules include action independent of phage host range (i.e., efficacy against bacterial strains that may be resistant to the parent phage) (Rodríguez-Rubio et al., 2016), flexible formulation, and favorable safety profiles. However, challenges persist: Phage-derived peptides are susceptible to degradation by endogenous proteases, accompanied by short half-life and poor *in vivo* stability. In contrast, antibody molecules possess high stability but have large molecular sizes and limited tissue penetration, leading to low delivery efficiency in complex environments such as solid tumors and biofilms (Vadevoo et al., 2023). Furthermore, some exogenous proteins may trigger immune responses, accelerate in vivo clearance and impair the efficacy of repeated administration (Washizaki et al., 2024).

## 7 Translational challenges and future perspectives

### 7.1 Technical bottlenecks

Host Range Specificity: Natural phages often have narrow host ranges (Holtappels et al., 2023). Its infection process relies on the specific recognition between tail fiber proteins or receptor-binding proteins (RBPs) and bacterial surface receptors such as lipopolysaccharides, capsules and membrane proteins (Cho et al., 2025; Deng et al., 2026). Accordingly, structural differences in receptors among various bacteria restrict the host range of phages. In addition, bacteria can resist phage infection through receptor mutation, restriction-modification systems and CRISPR-Cas immune systems (Baca et al., 2025; Wu et al., 2026; Liu et al., 2026). Current mainstream strategies include engineering modification of tail fibers or receptor-binding proteins (RBPs), optimizing phage genomes using CRISPR technology, and screening broad-spectrum mutant strains through directed evolution (Burke et al., 2025; Fernbach et al., 2025; Zhang S. et al., 2025). Among them, tail fiber modification and directed evolution are the most common approaches to broaden host ranges, which can enhance the infectivity of phages against drug-resistant bacteria and various bacterial strains. However, their genetic stability and long-term adaptability in complex environments still require further verification.

Pharmacokinetics and Delivery: Bacteriophages are subject to immune clearance (e.g., by reticuloendothelial system) and physicochemical inactivation. Strategies to enhance bioavailability include localized administration (topical gels, wound dressings, intramammary or intraperitoneal infusion) (Dąbrowska, 2019); formulation approaches (microencapsulation for oral delivery) (Pereira et al., 2023); and surface modification (e.g., PEGylation) to extend circulation half-life (Giorgi et al., 2014).

Immune Modulation Duality: Phages can act as potent immune activators as vaccine adjuvants but may also exhibit anti-inflammatory effects (Cianci et al., 2025; Souza et al., 2023). Critically, their ecological impact on commensal microbiota requires careful evaluation. Some studies hold that phages can specifically eliminate pathogenic bacteria, thereby alleviating gut microbiota dysbiosis caused by broad-spectrum antibiotics (Xu et al., 2026). Nevertheless, other studies have found that phages targeting key commensal bacteria may disrupt the balance of intestinal microecology. For instance, in gnotobiotic mouse models, phage cocktails against protective commensal bacteria such as *Escherichia coli* and *Enterococcus faecalis* reduced their abundance, while increasing host susceptibility to *Salmonella Typhimurium* infection, suggesting that phages may impair colonization resistance (von Strempel et al., 2023). Hence, the long-term ecological effects of phages on microbiota and their impacts on host immune homeostasis require further systematic investigation.

Biosafety and Regulatory Science: Key concerns include potential for temperate phages to mediate lysogenic conversion through transfer of virulence or antibiotic resistance genes (Anastassopoulou et al., 2025), environmental fate of engineered phages (Pires et al., 2016), and lack of globally harmonized regulatory frameworks for live phage biologics (Faltus, 2024). Existing regulatory pathways for veterinary medicinal products, magistral preparations, and feed additives were not originally designed for phage-based products, creating classification ambiguity and manufacturing cost challenges (Antoine et al., 2025).

### 7.2 Manufacturing and quality control

Large-scale production and quality control of engineered bacteriophages lay an important foundation for their clinical translation. Studies have shown that factors such as host bacterial status, culture conditions and purification processes may lead to batch-to-batch differences in phage titer and infective activity, thus affecting biological consistency (Yang et al., 2025; Modafferi et al., 2025).

In addition, most engineered bacteriophages are derived from Gram-negative bacterial systems, and their preparation is frequently accompanied by residual endotoxins including lipopolysaccharide (LPS). Accordingly, endotoxins need to be eliminated via chromatography and ultrafiltration to improve biosafety.

From the regulatory perspective, engineered bacteriophages must also comply with Good Manufacturing Practice (GMP) specifications, covering quality control requirements concerning sterility, genetic stability, purity and batch consistency. Overall, establishing standardized production and quality evaluation systems remains a major challenge for the clinical application of engineered bacteriophages.

### 7.3 Future perspectives

CRISPR-Phage Synergy: CRISPR-Cas systems enable precise phage genome engineering and phage-delivered CRISPR antimicrobials for sequence-specific targeting of pathogens or resistance genes (Roberts et al., 2025).

Phage-Nanomaterial Hybrids: Conjugation with nanoparticles can enhance phage stability, enable controlled release, and facilitate combination therapies (e.g., phage with photothermal agents) (Qi and Tay, 2025).

Synthetic Phage Genomics: *De novo* synthesis and modular assembly of phage genomes will enable “designer phages” with customized host ranges, regulated lysis cycles, and programmed multi-enzyme expression (Lenneman et al., 2021).

AI-Driven Phage Discovery: Machine learning models trained on phage genome, host range, and clinical outcome datasets will accelerate prediction of effective phage cocktails and in silico design of novel therapeutics (Doud et al., 2025).

Integrated Farm Health Systems: A holistic approach could combine population-level prophylaxis (Bianchessi et al., 2024), individualized precision therapy (rapid diagnostics followed by tailored phage cocktails) (Pirnay et al., 2024), and environmental biocontrol (phages to decontaminate facilities or degrade extracellular resistance genes) (Xue et al., 2024).

Long-term advances in synthetic and delivery technologies favor multifunctional engineered phages, yet their widespread application is hampered by limited host range, poor *in vivo* pharmacokinetics, biosafety concerns, immature manufacture and fragmented regulations. Though promising for precision therapy, further standardization of production, safety assessment and field trials is required to resolve existing bottlenecks prior to commercial rollout.

## 8 Conclusion

Bacteriophages constitute a programmable, multifunctional platform for controlling bacterial infections in animals. Their defining features—precision targeting, self-amplification, immunomodulatory capacity—directly address core limitations of conventional therapies including antimicrobial resistance and biofilm-associated chronic infections.

The evolution from natural lytic agents to engineered theragnostic platforms exemplifies the field’s transformative potential. Through synthetic biology, phages can be rationally designed to simultaneously deliver antigens, edit bacterial genomes, degrade biofilms, and reprogram local immunity—integrating therapeutic and prophylactic functions into single interventions.

Realizing this potential requires coordinated interdisciplinary efforts to overcome translational barriers: host range, pharmacokinetics, immune interactions, large-scale manufacturing, and regulatory harmonization. As research continues to elucidate tripartite interactions among bacteriophages, bacterial hosts, and immune systems, phages have the potential to become valuable components of the One Health arsenal, provided that current translational challenges are adequately addressed.
